# Abilify Maintena 400 mg (aripiprazole once-monthly), two-injection start (TIS) regimen: the experience of the Psychiatric Unit (SPDC) of Rimini

**DOI:** 10.1192/j.eurpsy.2024.1446

**Published:** 2024-08-27

**Authors:** M. P. Rapagnani, L. Veronesi, M. Magnani, F. Sartini

**Affiliations:** Psychiatric Unit, AUSL della Romagna, Rimini, Italy

## Abstract

**Introduction:**

The single-injection start regimen for aripiprazole once-monthly 400 mg (AOM 400) in patients with schizofrenia requires a single intramuscular injection in the gluteal or deltoid site and 14 days of concurrent oral therapy. Based on a population-pharmacokinetic model, the European Medicines Agency and Canada has recently approved a simplified starting strayegy of aripiprazole once a month with single-day regimen of two injections at separate gluteal and deltoid injection sites, together with a single 20 mg dose of oral aripiprazole on the 1^st^ day.

**Objectives:**

The aim of the study is to evaluate the two injection start (TIS) regimen in inpatients in the Psichiatric Unit (SPDC) of the Hospital of Rimini.

**Methods:**

We retrospectively reviewed medical records of patients, from February 2021 to April 2023, that have more than 18 years, who received the newly approved 2-injection start regimen as part of their standard care, evaluating if exist changes in clinical indicators, safety and tolerability of this regimen.

We valuated retrospectively the days of hospitalization after the aripiprazole 400 mg TIS and the number of emergency room access, analyzing the “repository of AUSL della Romagna” and discharge letters and the “CURE” program of the Psychiatric Service of Rimini.

**Results:**

We evaluated 24 patients from February 2021 to April 2023, 11 male (45,8%), 13 female (54,2%); average age 37,95, average lenght of stay in hospital was 11,75 days. 10 patients with diagnosis of psychosis/schizophrenia (41,7%), 6 patients with bipolar disorder (25%), 4 patients with personality disorder (16,6%), 2 patients with substance induced psychosi (8,3%), 1 patients with delusional disorder (4,2%), 1 patient with schizoaffective disorder (4,2%). 6 patients had the two-injection start regimen in 2021 (25%), 13 patients in 2022 (54,2), 5 patients in 2023 (20,8%); 20 patients did not have admission in hospital after the TIS (83,3%), 4 patients had 1 or more admission after the injection (16,7%). 3 patients (12,5%) had accesses in emergency-room after Abilify Maintena. 15 patients (62,5%) continue therapy; 9 patients (37,5%) had suspended the injection for drop-out or because of change of therapy not correlated at adverse effects (1 female patient had suspended treatment after the two-injections due to pregnancy). Just 1 patient that continue Abilify Maintena 400 mg had 2 accesses in the emergency-room.

**Conclusions:**

The coadministration of 2 injections of 400 mg aripiprazole was not associated with safety concerns beyond those expected with a single-injection start regimen. From the study it appears that the long-acting therapy with Alibify Maintena 400 mg once-monthly helps to stabilize the patient to prevent hospitalization and accesses in emergency-room.

**Disclosure of Interest:**

None Declared

